# The Optimal Timing for Pancreatic Islet Transplantation into Subcutaneous Scaffolds Assessed by Multimodal Imaging

**DOI:** 10.1155/2017/5418495

**Published:** 2017-12-26

**Authors:** Andrea Gálisová, Eva Fábryová, Eva Sticová, Lucie Kosinová, Markéta Jirátová, Vít Herynek, Zuzana Berková, Jan Kříž, Milan Hájek, Daniel Jirák

**Affiliations:** ^1^MR Unit, Department of Radiodiagnostic and Interventional Radiology, Institute for Clinical and Experimental Medicine, Prague, Czech Republic; ^2^Institute of Biophysics and Informatics, First Faculty of Medicine, Charles University in Prague, Prague, Czech Republic; ^3^Centre of Experimental Medicine, Institute for Clinical and Experimental Medicine, Prague, Czech Republic; ^4^Department of Clinical and Transplant Pathology, Institute for Clinical and Experimental Medicine, Prague, Czech Republic; ^5^Department of Pathology, Third Faculty of Medicine, Charles University, Prague, Czech Republic; ^6^Diabetes Centre, Institute for Clinical and Experimental Medicine, Prague, Czech Republic

## Abstract

Subcutaneously implanted polymeric scaffolds represent an alternative transplantation site for pancreatic islets (PIs) with the option of vascularisation enhancement by mesenchymal stem cells (MSC). Nevertheless, a proper timing of the transplantation steps is crucial. In this study, scaffolds supplemented with plastic rods were implanted into diabetic rats and two timing schemes for subsequent transplantation of bioluminescent PIs (4 or 7 days after rod removal) were examined by multimodal imaging. The cavities were left to heal spontaneously or with 10 million injected MSCs. Morphological and vascularisation changes were examined by MRI, while the localisation and viability of transplanted islets were monitored by bioluminescence imaging. The results show that PIs transplanted 4 days after rod removal showed the higher optical signal and vascularisation compared to transplantation after 7 days. MSCs slightly improved vascularisation of the graft but hindered therapeutic efficiency of PIs. Long-term glycaemia normalisation (4 months) was attained in 80% of animals. In summary, multimodal imaging confirmed the long-term survival and function of transplanted PIs in the devices. The best outcome was reached with PIs transplanted on day 4 after rod removal and therefore the suggested protocol holds a potential for further applications.

## 1. Introduction

Intrahepatic transplantation of pancreatic islets (PIs) represents an alternative treatment for unstable type 1 diabetic patients [1], but the procedure is associated with partial damage of liver tissue [2, 3] and graft impairment due to blood-mediated inflammation, rejection, or hypoxia [4]. Recognition of these limitations has increased the interest in the search for alternative transplantation sites in order to avoid liver-specific obstacles, provide adequate space for the transplanted islet mass, establish an efficient vascular network, restore physiological blood glucose levels, and minimise direct contact with blood [5, 6].

Recent studies have focused mainly on islet encapsulation and immunoisolation, documenting high clinical relevance [7]. However, it has been reported that encapsulated islets lack proper access to vascular vessels, nutrients, and growth factors [8, 9]. Another method advocated is the use of porous scaffolds (which can be removed in the case of complications) as a solid support for transplanted islets in order to facilitate vessel and tissue ingrowth along with nutrient diffusion [10–14]. A nondegradable macroporous Silon mesh rounded into a scaffold has been shown to exhibit suitable properties following transplantation into the omentum and subcutaneously [15, 16]. The scaffolds are supplemented with plastic rods to create a cavity for tissue and vessel ingrowth. Because subcutaneous vascularisation is insufficient for islets, several approaches have been advanced with the aim of improving neoangiogenesis, such as the incorporation of vascular endothelial and fibroblast growth factors [17] and mesenchymal stem cells (MSCs) [10, 18]. Our previous studies have also confirmed the improvement of blood supply in scaffolds induced by MSCs [15, 19]. In other models, cotransplantation of pancreatic islets in conjunction with MSCs have resulted in improved graft revascularisation, graft survival, and better transplantation outcomes [20, 21]. Several studies have shown that a lower number of islets can be used to reach normoglycaemia in diabetic animals in the cases of cotransplantation with MSCs [21, 22]. Nevertheless, accurate timing of the transplantation steps (implantation of scaffolds, transplantation of MSCs/islets) is crucial in order to reach sufficient vascularisation and a proper level of tissue ingrowth prior to PIs transplantation [11, 23]. Our previous experiments showed that the best engraftment period for PIs in the case of macroporous scaffolds is between day 3 and day 9 after rod removal [19].

To reveal the optimal time point for PIs engraftment, two time schemes for subsequent transplantation of PIs were tested in this study on diabetic rats. We compared the syngenic transplantation of PIs on days 4 and 7 after rod removal (11 and 14 days after scaffold implantation, resp.) and examined the effect of MCSs on transplantation outcomes. We also assessed islet engraftment and vascularisation using long-term* in vivo* magnetic resonance (MR) and bioluminescence imaging. Finally, we evaluated graft function by glycaemia monitoring and an optimal time schedule for the transplantation steps was proposed.

## 2. Materials and Methods

### 2.1. Animal Model

The bioluminescent (LUC+) and nonbioluminescent (LUC−) litters used in this study were the progeny of genetically modified heterozygous Lewis rats with ubiquitous expression of a gene for the luciferase enzyme (Lew-Tg(Gt(ROSA)26Sor-luc)11Jmsk, National BioResource Project, Rat, Kyoto, Japan). The bioluminescent rats were used as donors of LUC+ pancreatic islets and their LUC− littermates served as either recipients of the transplanted syngenic islets or as donors of LUC− MSCs. All recipients were 10-week-old male rats weighing 250–300 g at the time of islet transplantation. Two or three rats were used as islet donors for one recipient. The rats were placed under general anaesthesia (ketamine 36 mg/kg and dexmedetomidine 0.08 mg/kg; Vétoquinol, France and Orion Pharma, Finland) during all surgical procedures.

Diabetes was induced by injection of Streptozotocin (60 mg/kg, Sigma Aldrich) dissolved in cold 3.8% sodium citrate (pH 4.5) into the peritonea of the overnight-fasted LUC− recipients. One week later, 24 animals with confirmed diabetes (a minimum of 18 mmol/L blood glucose over 3 consecutive days) underwent subcutaneous implantation of macroporous scaffolds in the abdominal region. The scaffolds were prepared by shaping a 0.3 mm thin nondegradable Silon monofilament mesh (ELLA-CS, Czech Republic) into a rounded form with a cavity inside. The scaffolds were implanted with long polytetrafluoroethylene rods, which completely filled the cavities, in order to prevent total obliteration of the devices in all animals. The rods were removed from all scaffolds one week after implantation and the cavities were left to heal spontaneously or with 10 million LUC− MSCs, which were transplanted by 30 G needle syringe injection. The cavities were closed using small polytetrafluoroethylene plugs.

**Figure 1:**
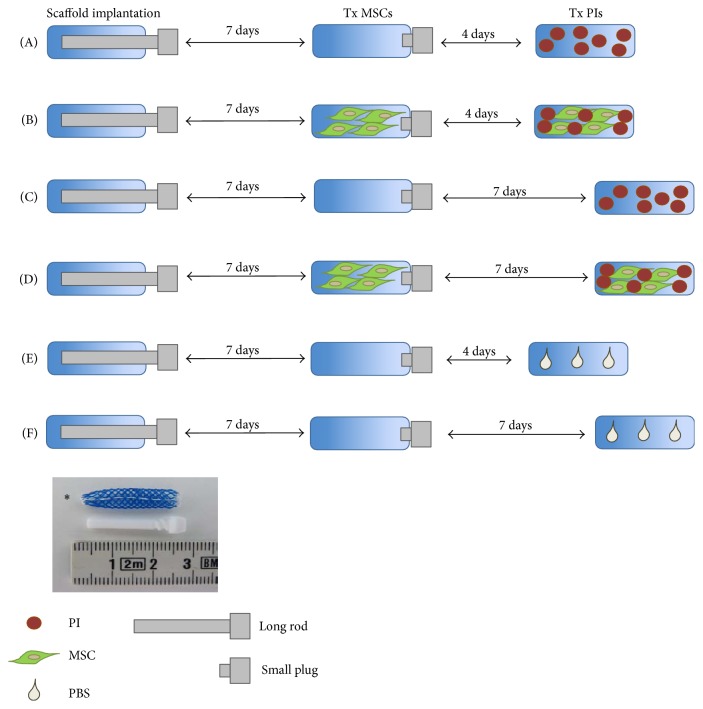
*Design of the experiment.* Scaffolds were divided into 6 experimental groups (A–F) according to the day of transplantation (Tx) of mesenchymal stem cells (MSCs) or pancreatic islets (PIs). Phosphate buffered saline (PBS) was added to the control scaffolds. The star indicates a photograph of a scaffold supplemented with a long polytetrafluorethylene rod.

The plugs were then removed and one thousand isolated LUC+ PIs were transplanted into the experimental scaffolds at two time points (4 or 7 days after rod removal) by injection using a syringe supplemented with a thin plastic tube for continuous injection. The scaffolds were divided into 6 experimental groups (*n* = 6 each): group A, islets were transplanted on day 4 after rod removal without MSCs; group B, islets were transplanted on day 4 after rod removal with MSCs; group C, islets were transplanted on day 7 after rod removal without MSCs; group D, islets were transplanted on day 7 after rod removal with MSCs; group E, control scaffolds containing PBS injected on day 4 were implanted into the animals with the scaffold group A; and similarly group F, control scaffolds containing PBS injected on day 7 were implanted into the animals with the scaffold group C. All control scaffolds were subjected to the same surgical procedures without transplantation of any cells or islets. Both experimental and control scaffolds were treated in the same way and after surgery the scaffolds were covered by skin and tightly sutured. The design of the experiment is shown in Figure 1.

To avoid the negative effect of hyperglycaemia on oxygen consumption in beta cells, a slow-release insulin pellet (Linplant Sustained Release Insulin Implants, LinShin Canada, Inc., Ontario, Canada; ≈2 U/day/implant for >40 days) was implanted subcutaneously in each animal at the time of scaffold implantation. The pellets were removed two weeks after islet transplantation in order to determine the effect of transplanted PIs on blood glucose levels. The body weights of animals were measured and blood glucose levels monitored on a regular basis using an automatic blood glucose metre for the duration of the whole 4-month experiment. Normoglycaemia was defined as a blood glucose level below 11 mmol/L (without fasting).

All animals were kept in a conventional breeding facility under a 12/12 light cycle regimen, with free access to pelleted food and water. The protocols related to the study were approved by the Ethics Committee of the Institute for Clinical and Experimental Medicine and the Ministry of Health of the Czech Republic in accordance with the European Communities Council Directive 86/609/EEC.

### 2.2. Isolation of MSCs

MSCs were isolated from the visceral adipose tissue of epididymal and perirenal areas in LUC− rats as previously described [15]. Briefly, fat tissue from the epididymal and perirenal areas was excised, washed twice with cold phosphate-buffered saline (PBS), and centrifuged (500 g, 5 min) after each wash. The rinsed tissue was digested by collagenase (Sevapharma, 1340 PZS/g, 1 mg/ml; Czech Republic) for 30 min at 37°C. Digestion was terminated upon the addition of ice-cold foetal bovine serum (Sigma Aldrich, USA) and the mixture was filtered through a 500 *μ*m mesh. The suspension was then centrifuged and washed three times (1000 g; 10, 10, and 5 min) in PBS with 1% antibiotic-antimycotic solution (AAS) containing penicillin, streptomycin, and fungizone (Thermo Fisher Scientific, USA). The tissue pellet was then resuspended in 3 mL of PBS with AAS and overlaid with 2 mL of Ficoll solution (1077 g/ml, Ficoll-Paque™ Premium, GE Healthcare Bio Science AB, Sweden). The cells in the interlayer were collected and then washed with PBS and cultured in DMEM low-glucose medium supplemented with 10% foetal bovine serum and 1% L-glutamine-penicillin-streptomycin solution (Sigma Aldrich, USA). The culture medium was replaced twice a week and the cells were subcultured for 2 weeks after isolation. Prior to transplantation, the cells were released from the bottom of the culture flask by trypsinisation, dissolved in cold PBS, quantified, and placed in a syringe.

### 2.3. Characterisation of Isolated MSCs

Tens of thousands of cells from each set were examined by fluorescent-activated cell sorting (FACS). The cells were incubated with anti-mouse/rat CD29 antibody (Biolegend, USA), phycoerythrin/CD44 antibody (Abcam, United Kingdom), PE-Cy™5 mouse anti-rat CD45 antibody (BD Biosciences, USA), anti-rat/mouse CD90.1 (Thy-1.1) antibody (E-Bioscience, USA), and anti-mouse endoglin/CD 105 antibody (R&D Systems, USA) for 20 min. The cells were then washed with FACS solution (PBS, 0.2% fish skin gelatin, and 0.01% sodium azide) and analysed by flow cytometry (BD FACSCalibur, BD Biosciences, USA). The analysis was performed using FlowJo 9.6.4 software (Tree Star, Inc., USA).

In order to assess the stem properties of isolated MCSs, cells were differentiated into chondrocytes, osteocytes, and adipocytes using a differentiation kit (RD Systems, USA). Briefly, cells seeded in a 24-well plate (adipocytes, osteocytes) or a 15 mL tube (chondrocytes) were cultured in adipogenic, osteogenic, and chondrogenic differentiation media, respectively, according to the manufacturer's instructions. After 21 days, adipocytes, osteocytes, and chondrocytes were detected using immunocytochemistry staining for FABP4, osteocalcin, and aggrecan, respectively.

### 2.4. Pancreatic Islet Isolation

LUC+ pancreatic islets were isolated from rats according to a standard isolation protocol [24, 25]. Donor pancreata (2-3 donor rats per recipient) were excised and digested using collagenase solution (Sigma Aldrich, USA). Islets were then separated from exocrine tissue using a discontinuous Ficoll (Sigma Aldrich, USA) density gradient (1.037, 1.069, 1.096, and 1.108 g/mL). Purified islets were incubated (37°C, 5% CO_2_ atmosphere) overnight in a CMRL-1066 culture medium supplemented with 5% HEPES buffer, 10% foetal bovine serum, and 1% penicillin/streptomycin/L-glutamine (Sigma Aldrich, USA). Islets were manually counted using a dissection microscope and collected in a small plastic tube connected to a syringe prior to transplantation.

### 2.5. Characterisation of Isolated Islets (Viability, Functionality, and Luciferase Expression)

The viability of islets before transplantation was evaluated after staining with the nucleic acid-binding fluorescent dyes, propidium iodide, and acridine orange (both Sigma Aldrich, USA) [26]. Ten handpicked islets were mixed with a 1 : 1 solution of propidium iodide (75 *μ*mol/L) and acridine orange (9 *μ*mol/L); after 5 minutes, 250 *μ*L of PBS was added to dilute the solution and the islets were examined under a fluorescent microscope. The ratio of the viable cells to all cells inside the islets was assessed for selected islets and expressed as an average percentage.

The functional potency of the islets was assessed using a glucose-stimulated insulin secretion test. Triplicates of 50 isolated islets were incubated in Krebs-Ringer bicarbonate buffer medium at low (3.3 mM), high (22 mM), and low (again) glucose concentrations (37°C, 1 hour). After each incubation, aliquots of the medium were removed and frozen at −20°C; insulin content was measured using the ELISA test (Mercordia, Sweden). The content of DNA in the sample was measured by PicoGreen Assay kit (Thermofischer Scientific, USA). The amount of insulin released upon glucose stimulation was assessed as the stimulation index, that is, the ratio of insulin values (normalised to DNA content) measured after stimulation and before stimulation.

Expression of the luciferase enzyme in isolated PIs was confirmed using an IVIS Lumina XR optical imager (Perkin Elmer, USA). Various amounts of isolated islets (50, 100, 300, 600, and 1000 PIs) were placed in the wells of a 6-well plate and imaged for 1 minute after the addition of 10 *μ*L D-Luciferin solution (30 mg/mL).

### 2.6. *In Vivo* Magnetic Resonance Imaging

The animals were anesthetised by inhalation of isoflurane (Isoflurane, Torrex, Vienna Austria) in air (5% for induction, 1% during the measurements). Animal body temperature was maintained using a heating system and breathing was monitored for the duration of all* in vivo* experiments. MRI measurements were carried out on a 4.7 T MR scanner (Bruker BioSpin, Germany) using a resonator coil with an internal diameter of 7 cm (Bruker BioSpin, Germany). Anatomical* T*_2_-weighted images were acquired using a fast spin echo sequence (repetition time TR = 3000 ms, echo time TE = 12 ms, turbo factor = 8, number of acquisitions NA = 4, time of acquisition TA = 5 min, and spatial resolution of 0.2 × 0.2 × 1 mm^3^). For dynamic measurements, a three-dimensional gradient echo sequence was used with the following parameters: TR = 10 ms, TE = 3.1 ms, spatial resolution = 0.2 × 0.4 × 0.7 mm^3^, 32 slices covering the scaffold volume, evolution delay = 2 s, temporal resolution of 40 s, and TA = 16 min. The MR contrast agent gadobenate dimeglumine (0.1 mmol/kg) was administered after the 8th cycle into the tail vein through a 24 G catheter. For analysis, regions of interest (ROI) in MR images were outlined around the internal diameter of each device using ImageJ software (version 1.46r, National Institutes of Health, USA). The area under the curve (AUC) of the DCE-MR signal was calculated within the first 160 s after administration of the contrast agent from the chosen ROI using GraphPad Prism 6.02 (GraphPad Software Inc, USA). The AUC was averaged from the 7 selected slices per time point. The rats were measured by MRI one day before PI transplantation and then on days 3, 7, 10, 14, 22, 34, and 60 after PI transplantation. All animals (with functional and non-functional grafts) were examined by MRI and bioluminescence.

### 2.7. *In Vivo* Bioluminescence Imaging

After MRI, the animals were placed in a dark chamber of the IVIS Lumina XR optical imager (Perkin Elmer, USA) in order to detect bioluminescence signals. Firstly, photographs were taken for anatomical coregistration of the bioluminescent source. Secondly, optical images were measured before and after intravenous administration of D-Luciferin dissolved in sterile PBS (50 mg/kg of body weight) with an exposure time of 1 minute, open aperture, and open emission filter. The images were acquired from a time series lasting 14 minutes, while the area under the dynamic time curve was calculated using GraphPad Prism 6.02 (GraphPad Software Inc, USA) in order to minimise the variability of D-Luciferin administration among the measurements. The bioluminescence examination was performed at the same time points as the MRI experiments until day 120 after PIs transplantation.

### 2.8. Histology

Four months after islet transplantation, all scaffolds were removed from the animals, fixed overnight in 4% paraformaldehyde (pH 7.4) at 4°C and embedded in paraffin blocks. Tissue sections (4 *μ*m) were cut and routinely stained with haematoxylin and eosin (H&E) and Verhoeff-Van Gieson elastin stain. Immunohistochemical detection of CD31 (rabbit polyclonal, Acris Antibodies GmbH, Germany) and insulin (mouse monoclonal, MU029-UC, Biogenex, USA) was performed on 4 *μ*m thick paraffin sections. The primary antibodies were applied overnight at 4°C. The CD31 antibody was detected by biotinylated goat anti-rabbit IgG (H + L) (Vector Laboratories, Burlingame, CA, USA), after which the sections were incubated with R.T.U. Vectastain Elite ABC Reagent (Vector Laboratories, USA) for 30 min. The Simple Stain MAX PO (MULTI) Universal Immuno-peroxidase Polymer anti-mouse, anti-rabbit Histofine (Nichirei Biosciences, Tokyo, Japan) was used to detect the primary anti-insulin antibody. Finally, the specimens were stained with the Dako Liquid DAB Substrate-Chromogen System (Dako) and counterstained with Harris's haematoxylin.

Microvascular density (MVD) assessment was performed on four serial sections from each scaffold. MVD was evaluated as the number of CD31-positive microvessels counted at a magnification of ×400 as previously described [19]. The results were expressed as the mean microvessel count with standard deviation.

### 2.9. Statistical Analysis

Statistical analysis was conducted using GraphPad Prism 6.02 (GraphPad Software Inc., San Diego, CA). The average DCE-MRI signals of the control and experimental scaffolds were compared per time point using the two-tailed Student's *t*-test or, for the whole examination, using the paired *t*-test. Comparison between three groups (with and without MSCs and controls) was performed by analysis of variance (ANOVA). The significance level was set at *p* < 0.05. Mean values and standard deviations are presented in the graphs. Coefficients of determination (*R*^2^) were calculated by regression analysis.

## 3. Results

### 3.1. *In Vitro* Examination of MSCs

**Figure 2:**
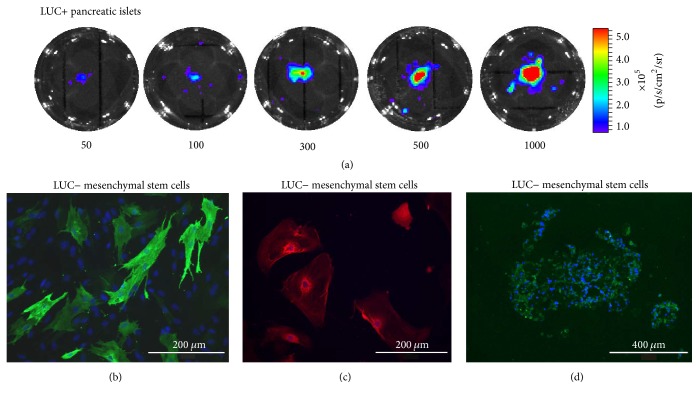
*In vitro characteristics of isolated MSCs and PIs.* Optical images of isolated PIs (a). The numbers below the images represent the amount of PIs in the well. Representative images of adipocytes (b), osteocytes (c), and chondrocytes (d) differentiated from isolated MSCs. The scale bar size is 200 *μ*m (b, c) and 400 *μ*m (d).

Flow cytometry confirmed stem characteristics of the isolated MSCs by the presence of specific molecules on the MSCs surface: CD29 in 95%, CD90 in 98%, and CD105 in 54%. Differentiation potency was confirmed by differentiation of the isolated MSCs into osteocytes, chondrocytes, and adipocytes (Figure 2).

### 3.2. *In Vitro* Examination of Pancreatic Islets

All isolated LUC+ pancreatic islets emitted photons following the addition of D-Luciferin due to luciferase expression (Figure 2). A linear relationship between the number of isolated PIs and the optical signal was found (coefficient of determination  *R*^2^ = 0.98). The viability of the islets assessed by staining with fluorescent dyes was >95% prior to transplantation. The mean of the stimulation index was greater than 5 in all groups.

### 3.3. Animal Model

No adverse macroscopic effects of scaffolds on the surrounding tissue were observed. There was no visible sign of inflammation, seroma, or macrophages migration on the day of scaffold retrieval and no perforation into skin or peritoneum during the whole examination. The scaffolds were removed from animals without causing of massive bleeding or other macroscopic damage. Scaffolds exploited higher level of vascularisation at the site closer to subcutaneous space compared to the site close to the muscle; therefore removal of the devices did not cause any harmful effect to the muscle tissue. The wound was easily sutured after scaffold removal.

**Figure 3:**
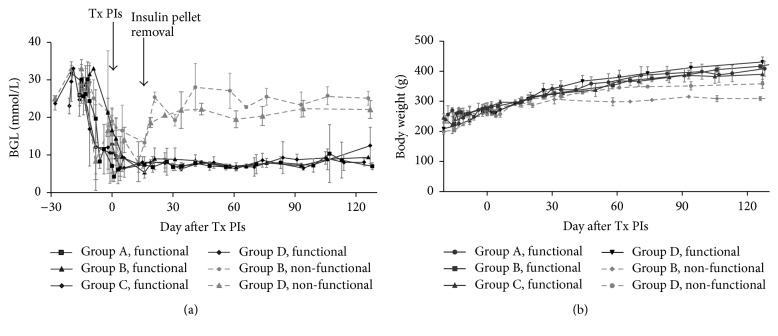
*Glycaemia and body weight of animals during the examination.* Blood glucose levels (BGL) of animals during examination show normalisation of glycaemia in groups A-D and diabetes reversal in groups B and D (a). The arrows show the day of pancreatic islet transplantation (Tx PIs) and the day of insulin pellet removal. Body weight changes of animals with functional and nonfunctional grafts (b). Nonfunctional grafts are represented by a dotted line.

Diabetes was reversed in all animals without MSCs; four animals from groups B (*n* = 2) and D (*n* = 2) remained hyperglycaemic (Figure 3(a)). The percentage of euglycaemic animals at the end of study was 100% in group A, 67% in group B, 100% in group C, and 60% in group D (one animal from the group D died during examination and it did not reach long-term normoglycaemia). Animals with nonfunctional grafts remained hyperglycaemic from the removal of the insulin pellet until the end of the examination. Normoglycaemia in animals with functional grafts was sustained for 4 months after removal of the insulin pellet. The body weights of animals with functional grafts increased regularly after PIs transplantation, while animals with nonfunctional grafts did not gain weight after PIs transplantation (Figure 3(b)).

### 3.4. MR Imaging

**Figure 4:**
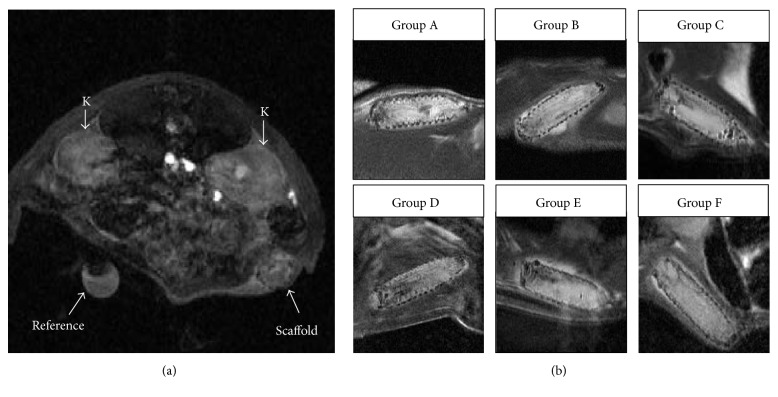
*Representative MR images of scaffolds*. Representative DCE-MR image of an animal with an implanted scaffold with MSCs and PIs (a) after contrast agent administration. The arrows indicate the kidneys (K), the scaffold, and a reference tube. Representative *T*_2_-weighted anatomical MR images of the scaffolds of different groups on day 7 after scaffold implantation (b).

*T*_2_-weighted MR images showed that scaffolds overgrew with connective tissue within the first two weeks. Air bubbles disappeared within one week after PIs transplantation. No anatomical differences between the scaffolds were found among the groups (Figure 4).

**Figure 5:**
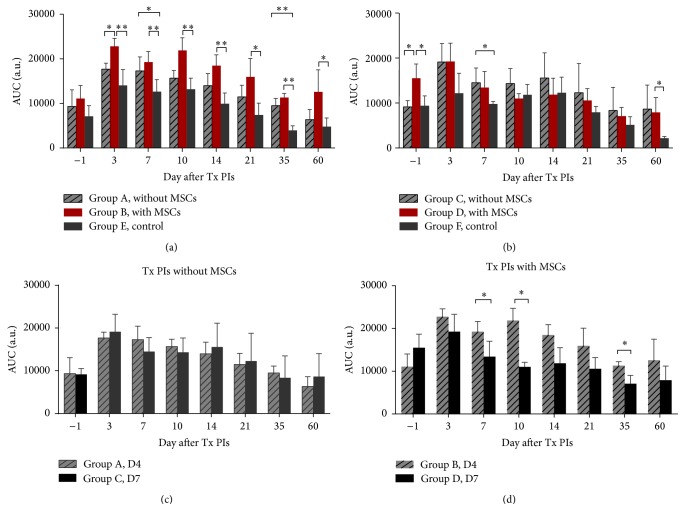
*DCE-MR analysis related to vascularisation.* Differences in AUC between scaffolds with and without MSCs and controls in animal groups with pancreatic islets transplanted on days 4 (a) and 7 (b) after rod removal. Comparison of pancreatic islet transplantation on days 4 and 7 after rod removal according to AUC values in scaffolds without (c) and with MSCs (d). ^*∗*^*p* < 0.05,   ^*∗∗*^*p* < 0.01.

Dynamic MR measurement revealed a peak in the AUC three days after PIs transplantation in all groups, after which AUC values continuously declined until day 60. Higher AUCs were observed in the scaffolds with the transplanted MSCs and/or PIs compared to the control scaffolds without any transplanted cells (paired *t*-test; *p* < 0.001 in groups A and B and *p* < 0.01 in groups C and D) (Figures 5(a) and 5(b)). Specifically, the difference between group B and controls (group E) was significant on all days following PIs transplantation, while the difference between groups A and E was significant on days 7 and 35 (Figure 5(a)) (unpaired *t*-test; *p* < 0.05). There was a significant difference between groups C and F on day 7 after PIs transplantation and between groups D and F on day (−1) and 60 (Figure 5(b)) (unpaired *t*-test; *p* < 0.05).

A significantly higher AUC was found in scaffolds supported with MSCs in comparison to scaffolds without MSCs (paired *t*-test; *p* < 0.001 in groups A and B and *p* < 0.01 in groups C and D). Unpaired *t*-tests revealed significance between groups A and B on day 3 (Figure 5(a)) and between groups C and D one day before PI transplantation (Figure 5(b)) (unpaired *t*-test; *p* < 0.05). The difference between all groups was not statistically significant after day 3.

Scaffolds with PIs transplanted without MSCs on day 4 and day 7 after rod removal showed similar AUC values (Figure 5(c)). Paired statistical analysis confirmed higher AUCs in the scaffolds with PIs and MSCs transplanted on day 4 after rod removal (paired *t*-test; *p* = 0.02). Unpaired statistical analysis revealed significant differences in AUC values on days 7, 10, and 35 (Figure 5(d)).

### 3.5. Bioluminescence Imaging

Optical imaging confirmed the presence of viable LUC+ PIs in the scaffolds for the duration of the whole examination, whereas no bioluminescence signal was detected in the control groups. Optical signals originating from the viable PIs reached their maximum within the first posttransplant week in all experimental groups (day 5 or day 7) and, after partially decreasing, remained stable for 120 days. In group C, the maximum bioluminescence signal (day 5) significantly differed from the signal measured on the last examination day (day 120) (*p* < 0.05). No significant difference was found between the maximum and the last measured signal in the groups A, B, and D.

**Figure 6:**
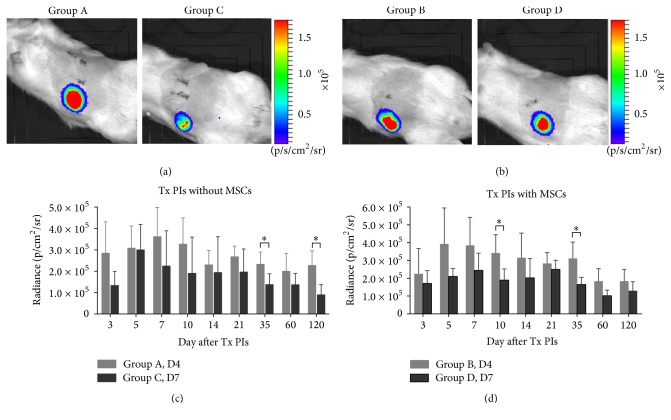
*In vivo bioluminescence imaging.* Representative* in vivo* bioluminescence images of pancreatic islets transplanted into scaffolds without (a) and with MSCs (b). Images show the scaffolds on day 7 after PI transplantation. Differences between optical signals originating from pancreatic islets transplanted on day 4 and day 7 after rod removal without (c) and with MSC support (d). D4 and D7 refer to the day after rod removal. ^*∗*^*p* < 0.05.

Pancreatic islets transplanted on day 4 after rod removal showed the higher optical signal regardless of MSC presence compared to transplantation on day 7 after rod removal (Figure 6). There was a significant difference in optical signals between groups A and C on days 35 and 120 (Figure 6(c)) and between groups B and D on days 10 and 35 (Figure 6(d)). Paired *t*-test analysis confirmed higher bioluminescence originating from PIs transplanted on day 4 compared to day 7 after rod removal (*p* < 0.0001).

There was no significant difference between the groups (with or without MSCs) transplanted at the same time point (*p* > 0.05 for all groups).

### 3.6. Histology

**Figure 7:**
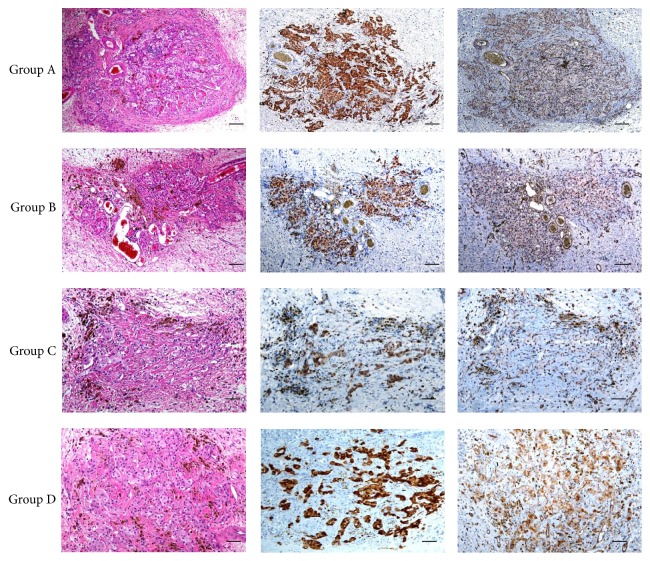
*Histology analysis*. Representative images of transplanted pancreatic islets in scaffolds stained with haematoxylin/eosin (H&E) and immunohistochemically with primary antibodies anti-insulin and CD31. Viable islets containing insulin positive cells were present in the scaffolds (H&E) in all groups A-D. Endothelial structures (CD31) were found in close proximity to the islets. Scale bars correspond to 100 *µ*m.

Histological analysis of the specimens stained with haematoxylin/eosin demonstrated the presence of viable islets in the central parts of all scaffolds (Figure 7, left row). Insulin deposits were found in all scaffolds (Figure 7, middle row). The microarchitecture of the islets was distorted by mild interstitial fibrosis with minimal inflammatory changes. Neovascularisation and fibrosis with haemosiderin deposits were also found in close proximity to the transplanted islets. Immunohistochemical staining with the anti-CD31 antibody showed higher microvascular density within the devices with transplanted MSCs (Figure 7, right row).

**Figure 8:**
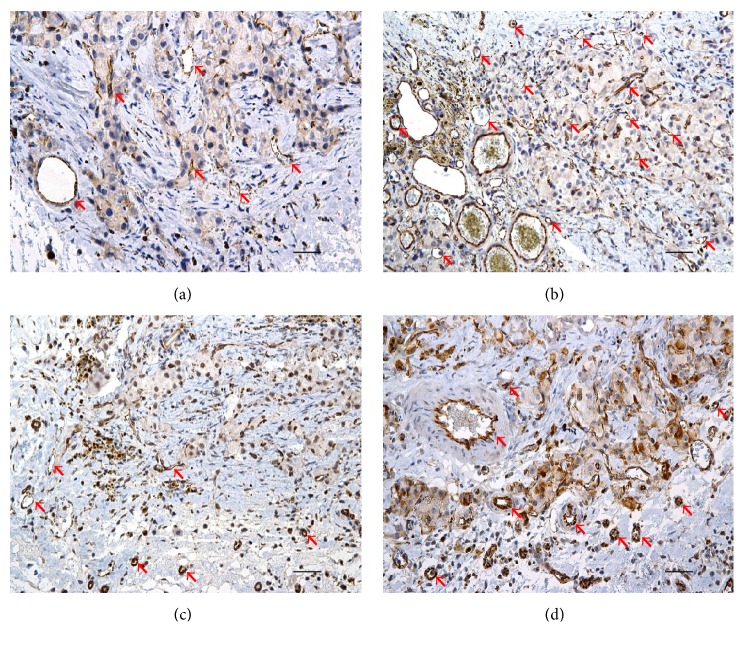
*Microvascular density analysis.* Representative images of CD-31 stained tissue sections from scaffolds. The arrows point vascular structures that were counted for MVD analysis in groups A (a), B (b), C (c), and D (d). Scale bars correspond to 50 *μ*m.

Quantitative analysis showed that the highest MVD was in the experimental scaffolds with pancreatic islets transplanted on day 4 after transplantation of MSCs (Group B). The mean microvessel count per ×400 field was 9.4 in group A, 17.7 in group B, 11 in group C, and 12.3 in group D. Representative images of CD-31 stained microvascular structures in scaffolds are shown in Figure 8. The results of DCE-MR examination (AUC) strongly correlated with the results of MVD analysis (*R*^2^ = 0.99).

## 4. Discussion

In this study, we assessed the optimal timing for transplantation of pancreatic islets into subcutaneously preimplanted polymeric macroporous scaffolds using multimodal imaging. Islets were transplanted into the scaffolds in short time after scaffold implantation, what represents a novel approach compared to the published ones [11, 12, 27, 28]. Since the subcutaneous area lacks adequate vascularisation, mesenchymal stem cells were transplanted into scaffolds prior to pancreatic islets in order to improve the local blood supply to a sufficient level for oxygen-demanding islets. It has been reported that MSCs secrete various proangiogenic substances, such as vascular endothelial growth factor (VEGF), platelet-derived growth factor, and angiopoietin-2 [29, 30], and topically stimulate growth of the vascular network [31–35].

Effect of MSCs on vascularisation was assessed by DCE-MR technique. The DCE-MRI signal reflects the rate of extravasation of a contrast agent within tissue. It can be quantified by calculating the area under the curve (AUC); its value is dependent on vascular permeability and perfusion, which are related to vascularisation. Our results show that the scaffolds with transplanted PIs and MSCs exhibited higher AUC values and were therefore more effective in forming the vascular network. This finding is in agreement with previously published data that report higher perfusion and vessel permeability in the same scaffolds with MSCs compared to controls [15, 19]. It is worth noting that in our study the difference between AUC values in the scaffolds with and without MSCs was significant only at some time points. This may have been caused by interanimal variability and the relatively low number of experimental animals. Also the amount of transplanted MSCs (10 million) could have only the modest effect on changes in vascularisation. The number of MSCs was chosen lower compared to the previous study [19] due to a limited inner space in the scaffolds and the required extra space for further transplantation of islets. Also in the first clinical study dealing with scaffold transplants it was discussed that the capacity of a device can be overwhelmed and then oxygen and nutrient exchange could be limited [27]. Because a positive effect of MSCs on PIs has also been observed after transplantation of a lower number of MSCs in other models with Lewis rats, for example, 3 × 10^6^ of MSCs in the omentum [22] or 1 × 10^6^ of MSCs in the kidney capsule [20], the chosen number of MSCs is appropriate in our opinion. Nevertheless, it is substantial that the results obtained by DCE-MR examination strongly correlated with the histological findings. Indeed, both analyses confirmed higher concentration of vascular structures in the scaffolds containing MSCs.

Moreover, a positive impact of MSCs on islet function and survival has been reported after coculture [23, 36] or cotransplantation of PIs with MSCs [20, 37, 38]. This beneficial effect of MSCs on islet survival is related to the suppression of inflammatory responses to transplantation itself and to allograft rejections [39, 40]. Some authors have also reported a positive influence of trophic factors released by MSCs such as VEGF [20], ciliary neurotrophic factor [41], Von Willebrand factor [20–22], and IL-6 [42] on islet survival. In our model, only viable islets produced a bioluminescence signal and therefore their viability can be assessed* in vivo *throughout the whole experiment. The results show that the bioluminescent signals originating from transplanted PIs were similar for the scaffolds with and without MSCs, while the normoglycaemia rate was even lower in the groups with MSCs. Therefore, we suggest that 10 million MSCs had only a negligible impact on syngenic graft survival and addition of MSCs also lowers islet function in this model. Given that a positive effect of allogeneic MSCs on PIs has also been reported [43–45], we predict that the influence of MSCs on transplanted islets could be more prominent in future allogeneic models. Also, nonfunctional islets in two scaffolds with MSCs may also indicate that MSCs transplantation may represent additional step threatening the success of pancreatic islet engraftment by another operation during the whole procedure. It is possible that an additional surgery (islet transplantation) led to reactions that harm MSCs and that slowed down immune reactions that could eventually protect the graft.

The main aim of this study was to identify the optimal timing for transplantation of pancreatic islets in scaffolds. Our previous experiments revealed that the best engraftment period for PIs is between 3 and 9 days after MSCs transplantation (10 to 16 days after implantation of the scaffold) [19]. Some other rat [11, 13] or mouse [12, 28] studies with transplant devices stated that 4 weeks are necessary for embedding the device by connective tissue, which should be free of inflammation and rich in new vessels. Also the first clinical islet transplantation into a prevascularised device was performed 1–4 months after device implantation [27], although the graft functionality was not reached. Here, our results indicate that a shorter time period (4 or 7 days after rod removal/11 or 14 days after scaffold implantation, resp.) is also suitable and that the tissue is well vascularised after this shorter period. Transplantation of PIs on day 4 after rod removal was found to be superior to transplantation on day 7 due to better vascularisation (assessed by DCE-MRI) and islet viability (assessed by bioluminescence). The islets transplanted on day 4 after rod removal emitted a higher optical signal, which reflects either the higher amount of surviving PIs or the better availability of a substrate for the bioluminescence reaction caused by higher blood supply. Nonetheless, bioluminescent signal of PIs transplanted on day 7 without MSCs significantly decreased between day 5 and the end of examination, what confirmed limited long-term survival of PIs under this transplantation condition. We hypothesise that on day 4 after rod removal, a layer of newly formed granulation tissue in the scaffolds created a matrix suitable for vessel growth and oxygen penetration. Over a longer period (7 days) the tissue could have become denser, restricting vessel and oxygen availability. In a novel site for PIs transplantation, consisting of a temporarily implanted catheter, an initial foreign-body response manifested by macrophage infiltration and neovascularisation was reported to be favourable for islet survival and function [46]. We contend that a similar process could have also manifested in our model; although we emphasize the importance of the use of a retrievable scaffold for possible removal in case of complications as rejection or inflammation. Also in a clinical study the devices were removed after certain period due to bacterial infection [27]. Another reason for using of a device is that transplanted islets are concentrated at one defined place, what is preferable for imaging, potential biopsy/histology, and for the delivery of drugs.

Although the effect of MSCs on islet engraftment was not prominent, the grafts were also functional and well-vascularised without the use of MSCs, which points to the strong influence of proper timing on islet engraftment. It is also worth noting that the transplanted mass was suboptimal and that the number of transplanted islets was lower compared to other scaffold models [11, 13], what is an important parameter for clinical application due to lack of donors. Nevertheless it should be noted that the results of preclinical studies might differ from clinical outcomes as it was the case of the first clinical trial with subcutaneous islet transplant device, where diabetes was successfully reversed in mice [12] but not in humans [27].

This study shows that islets transplanted on day 4 after rod removal reverted diabetes in all animals and normoglycaemia was maintained until graftectomy (4 months). These findings confirm the efficiency of the model presented here for type 1 diabetes treatment. Moreover, viable grafts and insulin deposits were confirmed by histology, which verify results obtained by other methods. The scaffolds tested were made from a clinically approved material and easily attainable adipose-derived MSCs were used, all of which may suggest the promising future applicability of these scaffolds in clinical practice.

## 5. Conclusion

In this study, we assessed the optimal timing for transplantation of pancreatic islets into subcutaneously implanted macroporous scaffolds. The scaffolds served as a suitable environment for the transplanted islets, which was confirmed by long-term reversal of diabetes, histology, and imaging methods. The addition of mesenchymal stem cells slightly improved local vascularisation but hindered islet engraftment and function in this model. Multimodal imaging indicated that the more suitable time for transplantation of pancreatic islets is day 4 after rod removal (rather than day 7) due to vascularisation and islet viability. We thus conclude that this optimised protocol of transplantation of pancreatic islets 4 days after rod removal holds a potential for further applications.
